
JOURNAL INFORMATION
==============================
NLM Title Abbreviation: Atlanta Med Surg J (1884)
NLM Journal ID: 101251623

Atlanta Medical and Surgical Journal (1884)

Publisher: Biomedical Journal Digitization Project

ARTICLE INFORMATION
==============================
PMCID: PMC9201864
PMID: 35829612
Article version: 1
Subjects: Correspondence

Correspondence

Warren W. C. M.D. London

Print publication date: 1899 Feb
Volume: 15
Volume Series: New Ser
Issue: 12
First page: 813
Last page: 814
License: This work is free of known copyright restrictions. For more information, please see PMC Back Issue Digitization.
License URL: https://creativecommons.org/publicdomain/mark/1.0/

==============================

### CORRESPONDENCE.

LONDON, January 1, 1899.

I will give briefly some observations I think will be of interest to the general practitioner as well as the specialist.

I find that the specialist of London is very conservative, not venturing into untried methods.

In acute middle-ear inflammation, at the onset of the attack, Dr. Dundas Grant treats by application of either hot fomentations or by irrigations with boiled water as hot as can be borne, either plain or normal salt or boric acid 2 per cent., together with instillations of sedative drop of cocain or opium or a small quantity of chloroform on a pad of cotton over the ear.

If pain is severe leeches to the tragus. If membrane is bulging a free incision should be made in posterior inferior quadrant.

In general aperients are advisable. Tincture aconite in small doses often repeated, phenacetine, etc.

Suppurative inflammation of the middle ear is an exaggerated form of acute catarrh, and should be treated similarly; but if there be deep-seated pain, especially over the mastoid, cold should be applied but not continued longer than forty-eight hours, as the more important symptoms are masked by which you are to judge of the necessity of operative interference. In all these cases you should suspect adenoids as being a factor in its causation, and when found removed after the acute subsidence. My attention was especially called to the fact of the general omission of specialists to look for adenoids in adults. I have seen a number of adults operated on with quite an improvement not only to their hearing but also to their general condition. While the adenoids are very small an acute coryza causes them to enlarge three or four times their dormant size, and in this way setting up the irritation that frequently causes the middle-ear involvement.

Tubercular laryngitis is receiving its share of attention in London, and the best results, according to Mr. Lenox Brown, are obtained before the ulcerative stage sets it by the intra-tracheal injections of 50 per cent guaiacol in almond oil, either in the hyperemic or anemic form.

After ulceration, cocainize thoroughly and curette every point of ulceration, and rub full strength lactic acid in with a rigid brush every two days. The curettage is to be repeated before each application of lactic acid.

Internal administration of guaiacol carb. or ferri and manganese in anemic persons. W. C. Warren, M.D.

---

Closure of Vesico-Vaginal Fistula Following Vaginal Surgery. (Chas. P. Noble, Philadelphia. Read before Philadelphia Obstetrical Society, October, 1898.)

Dr. Noble reports a case where a vesico-vaginal fistula followed vaginal hysterectomy for carcinoma of cervix. The opening into the bladder easily admitted three fingers, through the vesico-vaginal septum, the fistulous opening extending up to posterior wall of the vagina above the level of the vagina. The right edge of the vaginal wound was infiltrated with cancer, rendering the operation technically most difficult, and we would believe unjustifiable. It was necessary to dissect the bladder loose from the vagina in front and also from the subperitoneal connective tissue, as well as from the broad ligaments about the insertion of the ureters. This was difficult on account of the fixed condition of the parts, and from the fact that the vagina was contracted by post-climacteric atrophy. The fistula was closed with a fine running catgut suture reinforced with interrupted silkworm gut. The blander was drained with a Sims self-retaining silver catheter. The sutures were removed on the tenth day, but leaking continuing, the catheter was used for five weeks, resulting in perfect closure.

Noble reports two other similar cases showing the value of prolonged drainage of the bladder in the closing of fistula, in one of which the bladder was invaded by the malignant growth and was opened with a curette. A drainage catheter resulted in closure of the opening in ten days.—*McLaren.*
